# Clinical Characteristics and Outcomes of Limb Fractures in Saudi Children

**DOI:** 10.7759/cureus.56568

**Published:** 2024-03-20

**Authors:** Lamia Aldhbiban, Fai Alhoshan, Raghad Alomari, Shahad A Almatrafi, Yousef Alanazi, Samir Alsayegh, Haifa Y Alfaraidi, Ayman H Jawadi, Fahad N Aljuraibah

**Affiliations:** 1 Department of Pediatrics, King Abdullah Specialist Children’s Hospital, King Abdulaziz Medical City, Ministry of National Guard, Riyadh, SAU; 2 College of Medicine, King Saud Bin Abdulaziz University for Health Sciences, Ministry of National Guard Health Affairs, Riyadh, SAU; 3 College of Medicine, King Abdullah International Medical Research Center, Ministry of National Guard, Riyadh, SAU; 4 Department of Pediatric Surgery, King Abdulaziz Medical City, Ministry of National Guard, Riyadh, SAU; 5 Department of Pediatrics, Majmaah University, Al Majma'ah, SAU

**Keywords:** fracture healing duration, fracture predictors, fracture healing factors, fracture healing, pediatric fractures

## Abstract

Background: Children’s bones are at high risk of fracture as they grow. The clinical characteristics of fractures in children differ from those in adults. Studying fractures in healthy children is critical for identifying cases of fragility fractures. The aim of this study was to assess the clinical characteristics of limb fractures as well as clinical indicators of fracture healing outcomes in healthy Saudi children seen in an emergency room.

Methods: A retrospective review of the treatment course of all pediatric fractures and related factors treated at King Abdullah Specialist Children’s Hospital (KASCH) in Riyadh between 2016 and 2018 was conducted. Children with a primary bone disorder or chronic comorbidities known to affect bone health were excluded.

Results: The study included 143 patients (mean age ± SD = 8.23 + 3.76 years), and 71% (n = 102) were males. Motor vehicle accidents (MVAs) were the most common mechanism of injury, accounting for 50 (35%) cases, followed by fall injuries, sports injuries, and pedestrian accidents at 45 (31.4%), 16 (11.2%), and 13 (9.1%), respectively. A total of 178 fractures were reported, with the femur (n = 75, 42.1%) being the most common of the reported fracture sites, followed by the forearm (n = 44, 24.7%). The most common type of fracture was transverse fracture (n = 96, 54% of patients). Vitamin D levels were measured in 53/143 cases. Of these, vitamin D deficiency was found in 38 (71.7%) patients. The average time for fracture healing was 32.9 ± 30.2 weeks. The mechanisms of injury, including MVAs and sports injuries, as well as femur and forearm fractures, were clinical factors that were independently associated with a longer duration of fracture healing time (p < 0.001), but age, gender, and vitamin D status were not associated with that outcome.

Conclusion: MVAs and fall injuries were the most common causes of fracture in our patients. MVAs and sports injuries were associated with prolonged healing time. Large prospective, multicenter, or field studies may be required to further explore clinical characteristics, outcomes, and environmental factors.

## Introduction

Fractures in children are a common presentation. Prior to adulthood, it is estimated that half of all healthy children will sustain at least one fracture [1]. Fractures account for 10-25% of all pediatric injuries [2]. Boys sustain fractures more than girls, and the fracture rate is highest during puberty [1,3,4].

Previous epidemiological studies of fractures in children have demonstrated that fractures in the upper extremities occur more frequently than in the lower extremities and that the forearm is the most common fracture site, with fall injuries being the most common mechanism of injury [5]. The pattern of fracture sites and the mechanisms of injury vary with age.

The incidence of fractures in children has increased over time. A population-based study found that the incidence of fractures in children increased by 13% between 1998 and 2007, which could be explained by changes in children’s activity patterns over time [6]. Factors in addition to high-risk physical activity, such as male gender, poor nutrition, increased body mass index (BMI), and low bone mineral density, may increase susceptibility to fractures in children [1]. Similarly, vitamin D deficiency may also increase fracture susceptibility in children since vitamin D plays an essential role in calcium homeostasis and skeletal mineralization, which may influence fracture rates and healing time in children [7,8].

Fracture healing in children is influenced by multiple factors that are either related to the injury itself or the child [9,10]. For example, cancellous bone tends to heal faster than cortical bone, and fractures that occur near open growth plates tend to heal faster. Factors related to the child include the patient’s comorbidities, nutritional status, age, and medication use [11-15]. Importantly, vitamin D plays an essential role in bone mineralization and remodeling. Moreover, it has been shown in adult studies that vitamin D deficiency can lead to a delay in fracture healing and the disruption of the healing and remodeling process [14].

The clinical characteristics of fractures, including the impact of vitamin D status on fracture patterns and factors influencing fracture healing time, have not been well studied in Saudi children. The aim of this study is to describe demographic features, clinical characteristics, and fracture characteristics, such as the mechanism of injury, fracture type, and site, as well as healing time and its influencing factors in children presenting to our specialized tertiary center in Riyadh, Saudi Arabia.

## Materials and methods

A retrospective review was conducted using data gathered from children below 14 years of age who presented with upper and lower limb fractures to the emergency department at King Abdullah Specialist Children’s Hospital (KASCH), Riyadh, Saudi Arabia, from 2016 to 2018. Children with a primary bone disorder or chronic comorbidities known to affect bone health were excluded. The clinical data collected included age, gender, fracture date, age at the time of fracture, mechanism of injury, number of fractures at the time of presentation, type of fracture, fracture location, and estimated healing time from the time of the fracture to complete healing. If a serum bone profile was available at the time of fracture, data regarding calcium, phosphate, alkaline phosphatase, and vitamin D levels were collected at the time of fracture. Vitamin D levels were considered sufficient if greater than 75 nmol/L, insufficient if between 50 and 75 nmol/L, and deficient if less than 50 nmol/L.

The study was approved by the Institutional Review Board of the King Abdullah International Medical Research Center (KAIMRC), Ministry of National Guard Health Affairs, Riyadh, Saudi Arabia.

The data were entered and analyzed using SPSS v21 software (IBM Corp., Armonk, NY). Descriptive statistics are presented as frequencies and percentages for the categorical variables and means and standard deviations (SD) are supplied for the numerical variables. The associations between categorical variables were assessed using chi-square tests. Mann-Whitney U and Kruskal-Wallis tests were used to compare continuous variables in paired and multiple unpaired samples, respectively. Pearson’s correlation test was used to compare pairs of continuous variables. A probability (p) value of < 0.05 was considered statistically significant.

## Results

**Figure 1:**
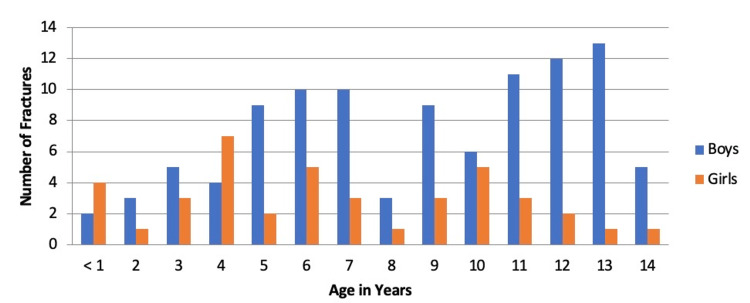
The frequency of fractures across each year in boys and girls.

Data were collected from 143 children with fractures (102 (71%) were boys). The mean age was 8.23 ± 3.76 years. The most frequent age of fracture was 13 years in boys and four years in girls (Figure 1). Eighty-six children (60%) had a single fracture, while 57 (40%) had multiple fractures. Motor vehicle accidents (MVAs), the most frequent cause of injury, were reported in 50 children (35%), followed by fall injuries, which were reported in 45 children (32%). Pedestrian accidents, the least common cause of injury, were reported in 13 (9%) children (Table 1).

**Table 1:** Demographic and baseline clinical characteristics of 143 children with fractures.

Characteristics	
Mean age (SD) - years	8.23 (3.76)
Gender, n (%)	
Male	102 (71)
Female	41 (29)
Frequency of fracture, n (%)	
Single	86 (60)
Multiple	57 (40)
Mechanism of injury, n (%)	
Motor vehicle accident	50 (35)
Fall	45 (32)
Sport injuries	16 (11)
Pedestrian injuries	13 (9)
Not documented	19 (13)

A total of 178 fractures were reported. The most common fracture site was the femur (75, 42.1%), followed by the forearm (44, 24.7%), tibia (26, 14.6%), fibula (18, 10.1%), and humerus (14, 7.9%). The least common fracture site was the ankle, which occurred in one child (0.6%). The majority of the fractures were displaced (128, 71.9%), with 50 (28.1%) non-displaced fractures. The most common types of fractures were transverse fractures (96, 54%), comminuted fractures (34, 19%), oblique fractures (25, 14%), and spiral fractures (17, 9.6%). The fractures were treated using a cast in 18.5% of the patients, while surgical intervention was required for 81.5% (Table 2).

**Table 2:** Clinical characteristics of 178 fractures.

Characteristics	
Fracture location, n (%)	
Femur	75 (42.1)
Forearm	44 (24.7)
Tibia	26 (14.6)
Fibula	18 (10.1)
Humerus	14 (7.9)
Ankle	1 (0.6)
Type of fracture, n (%)	
Transverse	96 (54)
Comminuted	34 (19)
Oblique	25 (14)
Spiral	17 (9.6)
Greenstick	6 (3.4)
Severity, n (%)	
Displaced	128 (72)
Non-displaced	50 (28)
Intervention, n (%)	
Surgery	145 (81.5)
Cast	33 (18.5)

Biochemical evaluation

A basic biochemical bone profile had been conducted in 70 of the 143 children. The mean serum calcium level was 2.24 ± 0.28 mmol/L, the mean serum phosphate level was 1.54 ± 0.31 mmol/L, and the mean serum alkaline phosphatase level was 251.5 ± 90.2 IU/L. Vitamin D levels were measured in 53 children; five had sufficient levels, while 10 and 38 had insufficient and deficient levels, respectively. There was a significant difference in vitamin D levels between genders, with more boys having low or insufficient levels than girls. The vitamin D level had a significant effect on forearm fractures; however, the other fracture sites did not show a significant difference. There was no significant difference in the mean age, mechanism of injury, and the frequency or severity of fractures between the vitamin D groups. Serum phosphate levels differed significantly between the vitamin D groups, with lower levels in the deficient group compared to the other group; however, serum calcium and alkaline phosphatase levels did not differ (Table 3).

**Table 3:** Factors associated with healing time.

Variable	Mean healing duration in weeks (± SD)	P-value
Age		0.192
Gender		0.360
Male	29.6 (24.2)	
Female	36.2 (28.7)	
Mechanism of injury		<0.001
Motor vehicle accident	40.6 (27.6)	
Fall	20.24 (20.3)	
Sport injuries	44.9 (28.5)	
Pedestrian accident	41.2 (9.7)	
Not documented	22.4 (19.6)	
Fracture location		
Femur	46.1 (27.6)	<0.001
Forearm	10.13 (6.5)	<0.001
Tibia	25.8 (13.4)	0.251
Fibula	25. (15.6)	0.720
Humerus	28.5 (26.1)	0.5814
Type of fracture		
Transverse	30.6 (25.2)	0.697
Comminuted	51 (32.9)	<0.001
Oblique	28.5 (13.8)	0.645
Spiral	19.9 (14.3)	0.159
Greenstick	2.85 (3.04)	0.302
Severity		0.122
Displaced	33.7 (2926.5)	
Non-displaced	25.1 (22.1)	
Intervention		0.012
Surgery	34.3 (27.3)	
Cast	18.9 (10.2)	
Vitamin D level		0.843

Association of healing time with clinical variables

The mean healing time of the fractures was 32.9 ± 30.2 weeks. The mechanism of injury was found to be significantly associated with healing duration (p = 0.029), with MVAs and sports injuries requiring notably longer healing times. The fracture site was also associated with healing duration. The femur as a fracture site was significantly associated with longer healing duration compared to other sites (p < 0.001), with a mean healing time for femoral fractures of 46.1 ± 27.6 weeks. On the other hand, the forearm was associated (p = 0.001) with shorter healing times (a mean healing time of 10.13 days ± 6.51 days) compared to other fracture sites. Treatment options were also significantly correlated with healing duration (p = 0.016). Tukey’s post hoc test revealed a significant difference between surgery and cast treatment (p = 0.023): the former required a significantly longer healing duration than the latter (34.3 ± 27.3 vs. 18.9 ± 10.2). No significant correlation with healing duration was found for gender, age, type of fracture, multiple fractures, displaced fractures, or vitamin D status (Table 4).

**Table 4:** Clinical characteristics of fractures in relation to vitamin D status in 53 children.

Variable	Sufficient vitamin D level (N = 5)	Insufficient vitamin D level (N = 10)	Deficient vitamin D level (N = 38)	P-value
Age, year, SD	5.4, 4.88	7.5	8.53, 3.19	0.168
Median	4 (2.5-9)	7.5 (91.65,11.25)	9 (6,11.25)	0.203
Gender				0.003
Male	0	8	28	
Female	5	2	10	
Mechanism				0.757
Motor vehicle accident	1	3	12	
Fall	4	5	16	
Sport	0	2	8	
Pedestrian	0	0	2	
Multiple fractures				0.333
Yes	3	5	28	
Displaced fracture				0.073
Yes	1	7	27	
Location				
Femur	2	6	18	0.709
Forearm	3	0	10	0.035
Tibia	0	4	7	0.158
Humerus	0	0	2	0.664
Type of fracture				
Transverse	4	6	18	0.343
Comminuted	0	2	6	0.580
Spiral	0	2	2	0.233
Oblique	0	0	7	0.204
Bone profile				
Calcium	2.42 ± 0.23	2.46 ± 0.15	2.25 ± 0.09	0.053
Phosphate	1.75 ± 0.13	1.61 ± 0.22	1.33 ± 0.18	0.038
Alkaline phosphatase	431 ± 285	347.3 ± 62	261.9 ± 72.7	0.171

## Discussion

This is a retrospective cohort study that reviews the healing course of children who sustained at least one fracture over a two-year period. The study found that boys sustained fractures more frequently than girls, especially around the pubertal age, whereas girls sustained fractures earlier. It has been found that MVAs were the most common mechanism of fractures and that the femur was the most common fracture site in our cohort. The mechanism of injury, fracture site, and fracture severity were the most influential factors in fracture healing in our patients.

In this study, fractures occurred more frequently in boys than in girls, which is consistent with most studies since boys tend to engage in more intense and competitive activities than girls [15]. While the peak age of fractures in both genders is around the age of puberty [1,4,15], this study found that fractures occur at a younger age in girls than in boys. Puberty is a critical time for bone growth; maximum bone accrual occurs during this period, but peak bone mass lags behind peak growth velocity, which occurs around the age of 11 years in girls and 13 years in boys, rendering the bone more fragile and increasing the risk of fractures, which may explain the high prevalence of fractures around puberty in children [16]. In our study, boys sustained fractures more frequently than girls at 13 years of age, whereas girls sustained fractures earlier, which might have been influenced by the high rate of MVAs in our study. Wang et al. reported that in children, injuries from MVAs decreased with age, while injuries from being struck by others and sprains increased with age [16]. The higher rate of fractures at an earlier age in girls needs to be further studied in a larger population.

Most epidemiological studies of fractures in children report that falls are the most common mechanism of injury [17,18], which is consistent with our findings that MVAs and falls are among the most common mechanisms of injury. In contrast to other epidemiological studies of fractures in children, our cohort has a higher rate of MVAs as a mechanism of injury. This can be explained by the fact that our center is one of the main trauma centers in the region. MVAs accounted for 83.4% of all trauma admissions in Saudi Arabia from 1984 to 1989, according to a systematic review of road traffic accidents [19]; however, no further research has been conducted to investigate the trend of road traffic accidents since then. Recently, stricter laws to reduce the incidence of road traffic accidents have been implemented, which may have resulted in a decrease in the incidence of MVAs, although this needs to be confirmed by further studies.

Given that MVAs were the most common mechanism of fracture, our study showed that the femur was the most common fracture site, which is consistent with other studies that investigated lower limb fractures following road traffic accidents and found that femoral fractures accounted for 31.5% of all fractures [20]. The upper extremities are the most common fracture site in children; specifically, the forearm and distal humerus are the most common fracture sites in childhood epidemiological studies [15,18,21]. The forearm was the second most common site of injury in our study. Aside from the high MVA rate, the severity of fractures in our cohort was high, with three-quarters of fractures being displaced. This may indicate that they were caused by a significant velocity impact, which may also influence the injury site, regardless of the mechanism of injury.

The radiological fracture healing process has been categorized into six stages, with stage one exhibiting a clear fracture line with no callus formation or bridging and stage six demonstrating complete union [22]. In our study, the average time for complete fracture union was 32 weeks. Complete radiological recovery of forearm fractures in our cohort was the shortest when compared to other fracture sites, where the mean healing time was 10 weeks, which was consistent with other pediatric studies that found the healing time for forearm fractures to be 2.5 months [23]. On the other hand, femoral fractures require more time to heal, with a mean healing time of 46 weeks, which is slightly longer than the 35 weeks reported by Grauberger et al. for pediatric femoral shaft fractures [24]. This disparity could be explained by the fact that our cohort included a high rate of MVAs and pedestrian accidents, which may be associated with a higher risk of vascular injury affecting the blood distribution at the fracture site, thus negatively impacting healing time. This was also evident in our study, where MVAs and pedestrian accidents were associated with longer fracture healing times compared to other mechanisms of injury.

Vitamin D levels, which were measured at the time of fracture for a subset of the cohort, showed no effect on fracture healing time. Vitamin D has an emerging role in fracture healing; however, studies on the effect of vitamin D deficiency on fracture healing time have yielded conflicting results [25]. We cannot conclude that vitamin D deficiency did not influence fracture healing time because our study was retrospective, vitamin D levels were not measured in the entire cohort, and vitamin D status was not documented at the fracture healing time as some children might be supplemented with vitamin D to correct the deficiency. In this study, 90% of the cohort who had a vitamin D level available had either a deficient or insufficient level of vitamin D, which is consistent with most national studies where vitamin D deficiency ranged from 60% to 96% in different age groups [26-29]. However, there was a significant difference in the rate of forearm fractures among different vitamin D levels, with the sufficient level group unexpectedly sustaining a high rate of forearm fractures, indicating that vitamin D status alone may not be associated with a high risk of fracture and that other factors, such as calcium and phosphate intake, must be considered. Interestingly, phosphate levels were significantly lower in the deficient group compared to the sufficient group, despite there being no significant difference in calcium levels between both groups. This can be attributed to the development of secondary hyperparathyroidism in response to calcium deficiency caused by vitamin D deficiency, thereby maintaining calcium levels at the expense of parathyroid hormone-induced phosphate loss in the urine.

A limitation of this study is that it is a retrospective and single-center study. Additionally, bone panel and vitamin D results were only available for a subgroup of the cohort. Moreover, other variables such as weight and height were not included in this study, as it was not well documented. However, one of its important strengths is that it includes many proposed factors that may influence fracture healing in a large cohort of Saudi children. A prospective study design that focuses on fracture healing is needed to better evaluate healing time and the factors affecting it.

## Conclusions

We have described the clinical nature of fractures in children and their healing time. MVAs were the most frequent mechanism of injury in our patients and the most influential factor in fracture healing time. This emphasizes the need for public awareness and the implementation of educational campaigns with the aim of reducing MVAs. Vitamin D deficiency, which is still prevalent in our community, was not well-presented in our cohort. Large prospective, multicenter, or field studies may be required to further assess clinical characteristics and outcomes as well as to examine the environmental factors affecting fractures in our children.
